# The significance of ovarian fibrosis

**DOI:** 10.18632/oncotarget.27822

**Published:** 2020-11-24

**Authors:** David A. Landry, Het T. Vaishnav, Barbara C. Vanderhyden

**Affiliations:** ^1^Cancer Therapeutics Program, Ottawa Hospital Research Institute and Department of Cellular and Molecular Medicine, University of Ottawa, Ottawa, Canada

**Keywords:** ovarian fibrosis, ovarian cancer, female reproduction, fertility, metformin

## Abstract

Ovarian aging is associated with significant changes in the structural organization of collagen, resulting in ovarian fibrosis. In many other tissues, fibrosis increases risks associated with tumorigenesis and metastasis. Thus, it is possible that ovarian fibrosis increases the risk of ovarian cancer by creating a microenvironment more permissive to tumor growth. In this research perspective, we review the impact of female reproduction on the development of ovarian fibrosis and the contributions of genetic and hormonal disruptions such as BRCA mutation, polycystic ovarian syndrome, and infertility to structural changes in the ovary and their relative risk of ovarian cancer. We also explore new fundamental questions in the field of ovarian fibrosis and possible prevention strategies such as metformin.

## INTRODUCTION

Increasing age in women has long been understood to be associated with the gradual loss of fertility due to the shrinking ovarian reserve. In a recent article published in "Clinical Cancer Research", McCloskey et al. [1] provided the first evidence that human ovaries undergo additional structural changes with age, including a significant change in the collagen architecture similar to fibrosis. Notably, we also reported preliminary data on the possible role of metformin in abrogating age-related ovarian fibrosis.

The general fertility rate in America has declined dramatically over the past decade [2]. With the modern life trend to delay motherhood, birth rates have increased for women between 35–44 years of age, raising the concept of ovarian aging as a new challenge to reproduction. Regrettably, a large portion of these women will have difficulties to conceive and will be recognized as sub-fertile or infertile. It is well known that fertility drastically declines after the age of 35, mainly by reducing the egg quality (reviewed in [3]). Mechanisms underlying ovarian aging and declines in fertility are well studied [4–6]; however, the relationship between ovarian fibrosis and fertility is not clear. In this current research perspective, we discuss more in depth the possible consequences of ovarian fibrosis on the risk for ovarian cancer with a specific focus on contributing factors associated with reproduction.

### Fibrosis in the aging ovary

In McCloskey et al., we reported that postmenopausal ovaries have higher coherence or collagen linearization (characteristic of organ fibrosis) compared to premenopausal ovaries that have an isotropic organization of collagen (characteristic of normal tissue). Further, age-associated ovarian fibrosis correlated with enhanced M2-like macrophage polarization, an observation that has recently been confirmed in mice and monkeys [7, 8]. Interestingly, postmenopausal women taking metformin have ovaries similar to premenopausal women with less apparent fibrosis, enhanced M1-like macrophage polarization and fewer features of chronic inflammation.

In women, many anatomical and physiological changes occur during each ovarian cycle, including growth of follicles (folliculogenesis), hormone production, ovulation and stromal tissue remodeling [9, 10]. Normal folliculogenesis is accompanied by an increase in pro-inflammatory cytokines which are associated with the acquisition of oocyte developmental competence during follicle growth, and allow the remodeling of the ovarian stroma to weaken the ovarian wall for follicular rupture and participate in wound repair after ovulation [11, 12]. Chronic inflammation is associated with development of fibrosis in other tissues [13, 14], and most chronic fibrotic disorders have a persistent irritant that will enhance fibrogenic cytokines and the deposition of connective tissue altering the normal tissue organization [15]. In the ovarian context, the repeated inflammation process of ovulation and wound healing over a reproductive lifetime could be that persistent irritant that leads to age-related ovarian fibrosis associated with higher permanent collagen deposition and increased risk to develop ovarian cancer.

Given the known contributions of general tissue fibrosis to tumorigenesis and metastasis and the 'seed and soil' hypothesis [16, 17], it is strongly anticipated that ovarian fibrosis is a risk factor for ovarian cancer. Moreover, fibrosis in cancer has been shown to increase tissue matrix stiffness, which promotes tumor progression and confers chemoresistance [16]. In a mouse model of ovarian cancer, cancer stroma with higher fibrotic gene expression was associated with a significantly lower survival rate [18]. As a matter of fact, not only ovarian stromal fibrosis is capable of creating a permissive 'soil' for metastasis, but fibrosis within ovarian tumors promotes cancer progression [18], supporting the urgent need for an ovarian fibrosis management strategy in aging women and ovarian cancer patients. In McCloskey et al., we provided new data on the possible effect of metformin on ovarian fibrosis and suggest that reducing the risk of ovarian cancer may be achieved by modulating ovarian fibrosis. A more detailed study evaluating how metformin can prevent and/or reverse ovarian fibrosis as a new potential non-invasive strategy to modulate age-associated fibrosis is warranted.

### Impact of ovarian fibrosis on fertility

Aside from surgical prophylactic oophorectomy-salpingectomy, it is not yet possible to efficiently prevent ovarian cancer; however, interrupting the ovarian cycle such as by pregnancies, lactation and/or use of oral contraceptives has been strongly associated with a reduced ovarian cancer risk, so much so that these measures are highly recommended to women at high risk [19, 20]. Pregnancies, breastfeeding and oral contraceptives all have in common their ability to prevent the ovarian cycle resulting in no antral follicle growth and no LH surge, subsequently preventing ovulation [10, 21, 22]. In McCloskey et al. [1], we found 2 of 11 postmenopausal women who are not taking metformin showed no signs of ovarian fibrosis. Unfortunately, the lack of information on the reproductive status of these women only allows us to speculate on the causes. Since parity, breastfeeding and oral contraception prevent ovulation, it is possible that postmenopausal women with no apparent fibrosis had had multiple pregnancies and/or took oral contraceptives for an extended period of time. Interestingly, recent studies demonstrated a significant effect of pregnancies on age-associated circulating cytokines, proposing that parity would enhance the immuno-surveillance and protect the ovary as a long-term 'imprint' against typical ovarian aging. Unfortunately, those studies did not explore if ovarian fibrosis was developed in multiparous mouse ovaries [23, 24]. However, based on those studies, we hypothesize that ovarian fibrosis is less apparent in women who have had multiple pregnancies and/or were on oral contraceptives, and that reduced fibrosis is central to the observed ovarian cancer risk reduction in these women. Future studies should evaluate the impact of reproductive decisions on the development of ovarian fibrosis in the longer term and their subsequent risk for ovarian cancer.

On the other hand, a large retrospective analysis demonstrated that *in-vitro* fertilization, including ovulation stimulation regimens, increased the incidence of ovarian cancer [25], although this remains controversial [26]. The potential impact of repeated ovulations on ovarian cancer risk opens a new window of association between fertility and ovarian fibrosis. In McCloskey et al. [1], we found 3 out of 11 premenopausal women with ovarian fibrosis. While the lack of information about their fertility history prevents us from drawing conclusions, it opens the door to proposing new hypotheses on the causes of premature ovarian fibrosis in premenopausal women. A recent study showed that more than 30% of infertile couples are diagnosed with unexplained infertility [27]. Moreover, several studies have reported that women with fertility issues are at increased risk of ovarian cancer [19, 28–30]. By deduction, would it be possible that some cases of infertility are directly caused by ovarian fibrosis which simultaneously also increases their risk for ovarian cancer? Would these women with unknown infertility issues be more at risk of ovarian cancer than women with known causes of infertility? Interestingly, there are several known pathologies characterized by ovarian fibrosis such as the ovarian chocolate cyst, polycystic ovarian syndrome (PCOS), and premature ovarian failure, all of which are more susceptible to infertility and early menopause (reviewed in [31]). As a common cause of infertility in women, PCOS is characterized by a persistent chronic inflammation in the ovaries but also by ovarian cysts, hyperandrogenism, inhibition of folliculogenesis and in some cases, anovulation [32]. Surprisingly, studies have shown conflicting data on the potential risk of PCOS ovaries to develop into ovarian cancer [33]. One long-term study showed no increase in mortality rates from ovarian cancer in women with PCOS compared to the general population [34]. On the contrary, a case-control study demonstrated a 2.5-fold increase in ovarian cancer risk in self-reported PCOS women [35]. More thorough studies are needed to better address the classes of PCOS and their respective risk for developing premature ovarian fibrosis or ovarian cancer. Based on the different grades and symptoms of PCOS, we hypothesize that ovarian fibrosis is lower in PCOS women diagnosed with anovulation (because of the lack of chronic ovulation), while PCOS women with higher chronic inflammation might be more susceptible to premature ovarian fibrosis and consequently be at higher risk of ovarian cancer. It is notable that women with PCOS are commonly treated with metformin to alleviate their metabolic symptoms; it remains to be determined whether metformin in this context also reduces ovarian fibrosis.

### Can ovarian cancer risk be reduced by inhibiting ovarian fibrosis?

While the risk of ovarian cancer in women with fertility issues, including PCOS, is not well established, women carrying a *BRCA* mutation are known to be at higher risk. Further, women carrying a *BRCA* mutation are at higher risk of developing ovarian cancer with a median age of diagnosis of 9 years earlier than non-carriers [36]. In addition to the consequences of *BRCA* mutation on ovarian cancer risk, there are various reports on its negative effect on female reproduction, most consistently its association with early menopause [37]. Since the *BRCA* gene functions in maintaining genomic stability and DNA damage repair [38], the impact of a lifetime of chronic inflammation and wound healing during the ovarian cycle in *BRCA* carriers may cause cellular damage that results in premature ovarian fibrosis leading to early menopause and increased risk of early ovarian cancer. In fact, our preliminary data suggest that BRCA ovaries are more fibrotic at an earlier stage in life compared to non-carriers. More work is needed to determine if *BRCA*-mutant ovaries have earlier onset of fibrosis than non-carriers and if metformin could be an appropriate strategy to reduce the ovarian cancer risk in these women.

Many ovarian diseases, mutations and natural processes, including PCOS, *BRCA* mutation, aging and ovulation may result in ovarian fibrosis. Our evidence that metformin may act as a preventive measure calls for further investigation. By preventing the ovarian cycle using oral contraceptives or pregnancies or delaying it by breastfeeding, it is possible to protect the ovaries on a long-term basis, which may directly lower ovarian cancer risk. Metformin potentially offers a new strategy to reduce ovarian cancer risk in high risk women who want to keep their reproductive choices open. This emerging field of study on ovarian fibrosis needs more work and faces a number of challenges. Obtaining human ovarian tissue to investigate the impact of reproduction, contraceptives, infertility and *BRCA1/2* mutations on ovarian fibrosis at different time points is not really feasible and alternative approaches such as functional imaging need to be explored. The use of molecular probes for imaging fibrosis in preclinical models of liver and lung fibrosis is a new and promising non-invasive assessment of organ fibrosis [39]. However, more work is needed to adapt and optimize those methods to small organs such as the ovaries. Without doubt, functional imaging of ovarian fibrosis will revolutionize the field and improve the diagnosis, prevention and treatment of ovarian fibrosis.
